# Developing sub-acute and non-acute Casemix classification: the Thailand experience

**DOI:** 10.1186/1472-6963-12-S1-I4

**Published:** 2012-11-21

**Authors:** Supasit Pannarunothai

**Affiliations:** 1 Centre for Health Equity Monitoring, Faculty of Medicine, Naresuan University, Thailand

## Abstract

**Invited speaker presentation:**

## Background

The needs for other modes of care apart from acute hospital care have long been realized but minimal integrated efforts have developed so far. Payment mechanism is a strong financing strategy to create favorable changes in the health systems.

## Objective

This session is to illustrate the rationale and progress of the developments of sub-acute and non-acute Casemix classification as a financing mechanism in Thailand.

## Method

Review of literature of the last five years is the main data collection.

## Result

The rehabilitative medicine has been an established discipline for over four decades in Thailand while psychiatry even longer. Complaints from health care providers of being underfunded activities have been around since the third revision of Thai Diagnosis Related Group since 2001. The fifth revision of TDRG set the goal of accomplishing other Casemix classifications apart from DRG for acute inpatient care, two Casemix systems for inpatient psychiatric and rehabilitative care were submitted to the National Health Security Office in 2011. The NHSO anticipated the difficulties of implementing the two new Casemix systems as initiative payment methods. Two separate streams of putting the Casemix products to the health systems were designed. Further follow-ups of these developments are needed to document knowledge generation related to health system outcomes via financing initiatives.

## Conclusion

Health finance and Casemix research need long-term commitment of a development team to refine results to find ways of implementing into health systems as well as to evaluate whether favorable changes happen.

